# Tissue Engineered Strategies for Skeletal Muscle Injury

**DOI:** 10.1155/2012/175038

**Published:** 2011-11-10

**Authors:** Umile Giuseppe Longo, Mattia Loppini, Alessandra Berton, Filippo Spiezia, Nicola Maffulli, Vincenzo Denaro

**Affiliations:** ^1^Department of Orthopaedic and Trauma Surgery, Campus Bio-Medico University, Via Alvaro del Portillo 200, Trigoria, 00128 Rome, Italy; ^2^Centro Integrato di Ricerca (CIR) Campus Bio-Medico University, Via Alvaro del Portillo 21, 00128, Rome, Italy; ^3^Centre for Sports and Exercise Medicine, Barts and The London School of Medicine and Dentistry, Mile End Hospital, 275 Bancroft Road, London E1 4DG, UK

## Abstract

Skeletal muscle injuries are common in athletes, occurring with direct and indirect mechanisms and marked residual effects, such as severe long-term pain and physical disability. Current therapy consists of conservative management including RICE protocol (rest, ice, compression and elevation), nonsteroidal anti-inflammatory drugs, and intramuscular corticosteroids. However, current management of muscle injuries often does not provide optimal restoration to preinjury status. New biological therapies, such as injection of platelet-rich plasma and stem-cell-based therapy, are appealing. Although some studies support PRP application in muscle-injury management, reasons for concern persist, and further research is required for a standardized and safe use of PRP in clinical practice. The role of stem cells needs to be confirmed, as studies are still limited and inconsistent. Further research is needed to identify mechanisms involved in muscle regeneration and in survival, proliferation, and differentiation of stem cells.

## 1. Introduction

Skeletal muscle injuries are common causes of severe long-term pain and physical disability, accounting for up to 55% of all sports injuries [1]. Contusions and strains are the most frequent muscle lesions, representing more than 90% of all sports-related injuries [2]. Mechanisms of muscle lesion can be divided into direct and indirect trauma. Direct injuries include lacerations and contusions, while indirect injuries include complete or incomplete muscle strains [3]. A muscle contusion takes place when a sudden, heavy compressive force is applied to the muscle [4]. A muscle strain occurs when an excessive tensile force is applied to the muscle leading to the overstraining of the myofibers up to a rupture near the myotendinous junction [5]. Muscle injuries can also result from a combination of these mechanisms. Finally, skeletal muscle can be damaged when compartment syndromes occur because of vascular and/or neurologic impairment [3, 6]. Injuries can counter the beneficial effects of sports participation because of the residual effects [7–19]. The associated morbidity, including painful contractures and muscle atrophy, can result in prolonged loss of activity and increased risk of recurrent injury [20]. In some instances, muscle injuries leads to inability of athletes to continue to practice sport [7].

Therefore, there is a need to improve skeletal muscle injury management. Conservative management is commonly accepted, according to the principle that “muscle injuries do heal conservatively”. It follows the RICE protocol (rest, ice, compression, and elevation). Other therapies include the local application of heat and passive motion exercises. Drug therapy typically consists of nonsteroidal anti-inflammatory drugs (NSAIDs) and intramuscular corticosteroids. 

Operative management is required only in selected patients, such as athletes with a large intramuscular hematoma, a complete strain of a muscle with no agonist muscles, a partial strain when more than 50% of the muscle belly is damaged, or persisting extension pain (>6 months) in a previously injured muscle [21].

As current therapy does not seem to obtain complete restoration of preinjury status, new biological therapies could represent interesting and more effective strategies to manage muscle injuries [22, 23]. Biological therapies include cell therapy, tissue engineering, and the administration of growth factors [24–26] with the goal of enhancing current therapies.

This paper provides an overview on current biological strategies for the management of patients with muscle injuries. The rationale behind these therapies and the best available evidence therapeutic options are reported.

## 2. Growth Factors

The healing process of the injured skeletal muscle is characterized by several bioactive molecules, including proinflammatory cytokines, transforming growth factor-beta (TGF-*β*) superfamily members, and angiogenic factors. For this reason, the growth factors and the cytokines represent a potential therapeutic option to improve the regeneration/repair process of injured skeletal muscles. These signaling molecules accelerate the regeneration of injured muscular tissue, providing a mitogenic stimulus activating myogenic precursor cells [27]. 

Each of these molecules shows specific biological activities. The transforming growth factor-beta (TGF-*β*) stimulates mesenchymal cell proliferation [28], promotes the proliferation of fibroblasts [29] and the biosynthesis of extracellular matrix, particularly type I collagen [30], regulates endothelial cell activity and angiogenesis [31], and inhibits satellite cell proliferation and differentiation [27]. Fibroblast growth factor (FGF) promotes proliferation of fibroblasts [32], stimulates satellite cells proliferation but inhibits their differentiation [33], and promotes the mitogenesis of mesenchymal cells [27]. Epidermal growth factor (EGF) stimulates fibroblasts migration and proliferation and regulates angiogenesis and extracellular matrix homeostasis [34]. The platelet-derived growth factor (PDGF) promotes the mitogenesis of mesenchymal cells and fibroblasts [35], induces proliferation of satellite cells, and inhibits the end stages of myoblast differentiation [36]. Vascular endothelial growth factor (VEGF) promotes endothelial cells mitogenesis and migration [37] and stimulates myoblast migration [38]. The neoangiogenesis plays a critical role in the healing process of muscle injuries. The new vessels sprout from the health tissue surrounding the lesion and provide the supply of oxygen, growth factor, and blood stem cell to enhance the regeneration process [39]. Thus, the restoration of vascular pattern in the injured area represents an early and necessary phase for regeneration and morphological and functional recovery of muscle tissue.

Based on the multitude of their biological effects, the clinical application of growth factors is affected by considerable side effects. An overexpression of growth factors such as TGF-*β* and FGF has been related to inhibition of myoblasts differentiation and muscle-fiber regeneration [33]. In addition, growth factors explain their stimulatory effect on both muscle cells and fibroblasts. Particularly, TGF-*β* is one of the most important growth factors related to scar formation during healing, and it seems to drive the differentiation of myogenic cells into myofibroblastic cells. For this reason, muscle-fiber regeneration and scar-tissue production can be considered two concomitant and competitive processes [40–57].

Also, the expression of growth factors is closely regulated by a large number of extracellular matrix (ECM) proteins, namely, the heparin sulfate proteoglycans and the small leucine-rich proteoglycans (SLRPs) [58, 59]. Several growth factors need to bind the heparan sulphate proteoglycans and the SLRPs to provide their biological effects. Thus, the application of growth factors to promote healing of the damaged muscle tissue should include the administration of these specific ECM molecules [13, 14, 16, 60–76]. 

To date, available data from experimental settings are contradictory. Some authors did not report any beneficial effects by the administration of FGF-2 [77] or overexpression of skeletal muscle specific isoform of IGF-1 (mIGF-1) at the injured region [78]. FGF-2, IGF-1, and nerve growth factors can promote muscle healing process, increasing resistance to tensile loading when compared to untreated muscles [79, 80]. Moreover, mouse myoblasts transduced with the IGF-I gene increase their growth rate and enhance the contractile force production of skeletal muscle substitutes consisting of hydrogel and IGF-I engineered myoblasts [81].

A combination of growth factors can be used to regulate the different process of regeneration of muscle tissue and scar-tissue production. Thus, the application of IGF-I combined with TGF-*β* allows to induce muscle regeneration, preventing the formation of a fibrous scar [82].

## 3. Platelet-Rich Plasma (PRP)

Platelet-rich plasma (PRP) therapy represents an interesting biological technique to provide tissue repair by inducing chemotactic, proliferative, and anabolic host cellular responses [83]. PRP is an autologous product consisting of bioactive agents derived from patients' own platelets [84–86]. Usually, PRP is administered by local injection of the PRP solution or the application of a PRP gel at the time of surgery [87].

Given the large amount of biological agents required for tissue repair, PRP could be an ideal biological autologous product providing a balanced combination of mediators able to improve the healing process. In clinical practice, the blood clot at the site of injury is replaced with a smaller volume of PRP solution or gel. The increased concentration of platelet at the site of lesion provides a higher concentration of healing bioactive factors than in physiological conditions. To date, PRP has been proposed for management of tendon [88–90], ligament [91, 92], muscle [93], nerve [94, 95], bone [96, 97], and joint injuries [98, 99].

The effectiveness of autologous conditioned serum (ACS) has been compared with Traumeel/Actovegin in a nonrandomized nonblinded pilot study (level III) on muscle strain injuries in professional sportsmen [93]. The ACS was obtained from whole blood, and it contained bioactive proteins including interleukin-1*β* (IL-1*β*), TNF-*α*, IL-7, fibroblast growth factor-2 (FGF-2), IL-1Ra, HGF, PDGF-AB, TGF*β*1, and IGF-1. Traumeel is a homeopathic formulation containing both botanical and mineral ingredients in homeopathic concentrations. Actovegin is a deproteinized calf blood hemodialysate consisting of a physiological mix of amino acids. Although both treatments were safe, the ACS allowed to reduce the time to full recovery and the amount of edema and/or bleeding at MRI images. 

These findings have been also confirmed in professional soccer players with muscle lesions varying for size and location [100]. Athletes were managed with activated pure PRP (P-PRP) injections. Full resumption of normal training activities was restored in half of the expected time compared to matched historical controls. The same leukocyte-free PRP preparation has been found effective to manage adductor longus strain in a professional bodybuilder [101].

ACS and PRP have been also evaluated in laboratory settings. ACS was compared with saline solution in a contusion injury model. ACS showed an earlier activation and/or recruitment of satellite cells, and an earlier fusion, with larger regenerating myofibers, compared with controls [102]. PRP increases proliferation of muscle cells, differentiation of satellite cells and synthesis of angiogenic factors in an *in vitro* setting [103]. PRP and leukocyte and platelet-rich plasma (L-PRP) have been also compared in a laboratory-controlled study using a muscle strain rat model. The authors demonstrated that PRP is more effective than L-PRP in terms of myogenesis enhancement and contractile function [104].

Although preliminary data are encouraging, there are some reasons of concern about PRP treatment. First of all, PRP could induce a fibrotic healing response in muscle tissues, by increasing local concentration of TGF. According to experimental data, TGF seems to be able to induce fibrosis in cultured muscle tissue [27]. Moreover, the effectiveness of PRP could be affected by leukocytes within the injected solution, because their enzymes (proteases and acid hydrolases) can damage muscle tissue [105]. 

Finally, several devices and systems are available for PRP preparation. Therefore, PRP products applied in several studies consist of a basic mixture of grow factors including different concentration of each single agent. Moreover, level I studies performed with adequate outcome measures and follow-up assessment are lacking. 

To date, no PRP formulation has solid evidence of effectiveness to heal muscle injuries. Pilot clinical studies indicate that PRP therapies may enhance muscle repair after strain or contusion. Moreover, laboratory data indicate the ability of several growth factors to enhance myogenesis. However, at present, there is no evidence to recommend or discourage the adoption of PRP in clinical practice [25, 26, 106–129]. Further research is required to standardize formulations (number of platelets and/or leukocytes) and administration regimens, including volume of injection and timing of treatment, to optimize PRP application for management of muscle injuries.

## 4. Cell Therapy

In the last decade, regenerative medicine and tissue engineering increased their role in management of musculoskeletal diseases. Transplantation of stem cells has been considered a new strategy to repair injured tissues [130–142]. Different areas of application have been explored, such as articular cartilage [143–146], bone [147–150], ligament, and tendon [151–155]. The expectations for future therapeutic strategies are great.

The idea of cell therapies for muscle regeneration has been developed from the observation that skeletal muscle has regenerative capacity [156–158]. Several studies have investigated the role of stem cells in muscle healing, showing their direct participation in tissue regeneration and their influence in healing modulation [30, 159, 160]. However, severe muscle injuries are characterized by concomitant activation of regenerative activities of the satellite cells and profibrotic activities of fibroblasts [161, 162]. 

The specific expression pattern of growth factors in the region of injury determines the dominant cell type in the wound healing process [30]. High levels of TGF-*β*3 are related to the activation of mesenchymal progenitor cells (MPCs) derived from traumatized muscle to promote wound healing after muscle injury [163]. On the other hand, high levels of TGF-*β*1 are related to activation of fibroblasts to produce disorganized extracellular matrix leading to fibrosis in the muscle tissue [159, 160]. The fibrotic tissue affects the ability of the satellite cells to repair the muscle tissue.

There are distinct subsets of myogenic cells. Muscle satellite cells (SCs) are localized under the basal lamina of muscle-fibers [164]. They respond to regenerative stimuli by proliferating to form myoblasts which, in turn, differentiate and fuse in multinucleated myotubes [165, 166]. Their capability to renew and to produce differentiated progeny suggests that they are the adult stem-cell population of skeletal muscle [167]. They are also known as Pax7+ cells, based on their expression of the muscle-specific paired box (Pax) transcription factor Pax7 [168]. However, SCs consist of heterogeneous cell population, including Myf5+ cells (90%) and Myf5-cells (10%) [169]. The first of them are committed to the myogenic lineage because of expression of Myf5 which is an initiator of myogenic differentiation [170].

Other stem cells have been identified. They are both muscle specific, such as mesoangioblasts and pericytes, skeletal muscle precursors (SMPs), muscle stem cells (MuSCs), side-population (SP) cells, and PW1-cells, and nonspecific, such as embryonic stem (ES) cell, amniotic fluid stem (AFS) cells, mesenchymal stem cells (MSCs), and mesenchymal cells from bone marrow [171]. They are able to contribute to muscle regeneration with different myogenic potential, but their potential is still undefined. Satellite cells seem to be sufficient for the regenerative need of damaged adult skeletal muscle *in vivo* [172]. The MSCs present a great migration potential toward the areas of induced muscle degeneration and undergo myogenic differentiation, providing regeneration of muscle tissue. The MSC transduction with transcription factors, such as MyoD, has been also investigated to enhance their potential of myogenic differentiation [173].

The properties of the stem cells in the muscle have been analyzed using animal models of muscle dysfunctions and injuries. Improved muscular structure has been observed in mice used as Duchene Muscular Dystrophy models treated with stem cells [174–176]. Better muscle regeneration has been obtained by the use of muscle-derived stem cells (MDSCs) in models of induced skeletal muscle injury [177]. MDSCs provide an improvement of muscle healing because of their ability to recruit capillaries and nerves into the injured region [177]. They are also able to differentiate directly into endothelial cells and cell types with neuronal characteristics [178]. For these reasons, muscle regeneration seems to be more powerful with MDSCs application compared with satellite cells application.

A model of hindlimb ischemia, analogous to exercise-induced compartment syndrome, showed potential benefit of injections of marrow-derived stromal cells in term of perfusion, fibrosis development, and atrophy [179]. Results from ongoing studies on MDSCs implantation after musculoskeletal contusion are awaited [162].

The role of stem cells in musculoskeletal disease needs to be confirmed. Studies are still limited, and many questions are still unanswered. Several issues should be taken into account, such as safety and efficacy, immunogenicity, and biochemical factors involved in survival and differentiation of stem cells. Further research is needed to identify mechanisms involved in muscle regeneration to exactly understand the therapeutic potential of stem cells.

## 5. Scaffolds

Regenerative medicine is a multidisciplinary approach to produce living, functional substitutes for restoration, maintenance or improvement of the function of damaged tissue or organ. Tissue engineering is a specific approach included in regenerative medicine field. The tissue engineering consists of association of three main elements: cells, factors or stimuli, and biomaterials [180].

Musculoskeletal tissue engineering aims to obtain functional replacement of lost or damaged bone, cartilage, skeletal muscle, and tendon/ligament [8, 15, 17–19, 22, 181–195]. In skeletal muscle injuries, tissue engineering represents a biological alternative for replacement of large tissue loss after severe damage.

Skeletal muscle tissue engineering could be performed by two different approaches: *in vitro* and *in vivo*. In *in vitro* tissue engineering, SCs from adult skeletal muscle are expanded and seeded on a 3D scaffold to produce a cell-biomaterial construct. After the differentiation of stem cells, the neo-tissue graft could be transplanted in the injured region. In *in vivo* tissue engineering, the isolated SCs are charged on a 3D scaffold carrier and promptly transplanted. Thus, the delivery of stem cells in the muscle lesion is obtained [171].

Efficient skeletal muscle regeneration is strongly related with features of biomaterials used to fabricate scaffold and with the regenerative potential of cells used for scaffold seeding. The source of cells used for scaffold seeding should be chosen based on the features of the damaged tissue. Cells can be autologous or allogeneic, including also stem cells where it is required.

The scaffold is a 3D-structure able to mimic the anatomical and biomechanical properties of the native tissue. The scaffold for muscle tissue engineering should be able to flex and stretch [196]. Moreover, they should be able to promote the alignment of myoblasts the assembly of myotubes. Nanostructured scaffolds are more efficient in promoting myotube assembly than microstructured scaffolds [197].

The biomaterials used to fabricate scaffold can be natural (like collagen) or synthetic (e.g., ceramics, polymers of lactic, and glycolic acid) and soluble or insoluble. Scaffold must have biocompatibility and biodegradability properties [198]. Biocompatibility is essential to prevent toxicity and immunogenicity biomaterial-related inducing the immune-response in the host muscle. Biodegradability allows gradual substitution of the scaffold by the newly formed muscle tissue. Moreover, the scaffold should integrate molecules or cells, providing a controlled delivery of growth factors, cytokines, plasmids, drugs, or other anabolic stimuli [199–202]. In skeletal muscle tissue engineering, biomaterials should support the myogenic process, providing a microenvironment which allows cell survival, proliferation and/or differentiation to repair, and/or regenerate the damaged tissue [9–11, 23, 203–221].

Both synthetic and natural scaffolds have been investigated for tissue engineering approaches to muscle regeneration. The polylactic-co-glycolic acid (PGA) is a synthetic biodegradable biomaterial showing appropriate rigidity and connection, appropriate for muscle tissue engineering. Constructs of myoblasts and polyglycolic acid meshes have been evaluated in a muscle regeneration rat model. Regenerate tissue-like structures have been found with aligned myoblasts along strands of polymer fibers. The PGA scaffold allowed the alignment of myoblasts and the assembly of myotubes, reproducing the organization of muscle fibres [222].

In the field of natural biodegradable biomaterials, different 3D scaffolds have been developed. Collagen scaffolds with parallel oriented pores have been used to reproduce the three-dimensional organization of skeletal muscle [223]. Permanent myogenic cells were infiltrated in these scaffolds and were cultured to induce their proliferation and differentiation [223]. The collagen scaffold with oriented pore structure showed the ability to induce skeletal muscle-like tissue regeneration with aligned multinucleated myotubes according to the orientation of pore structure [223]. In addition, cell-scaffold constructs were able to support mechanical forces generated in muscle tissue [223]. These results have been also found in an *in vitro* study in which a multilayered cultures of rat neonatal satellite cells in collagen 3D scaffolds were performed [224].

Fibrin is another natural biodegradable 3D scaffold used to obtain muscle regeneration. Three-dimensional fibrin matrix has been used as carrier to inject myoblasts in the injured muscle region of a rat model. The fibrin carrier induced no inflammatory reaction and allowed integration of myoblasts into host muscle-fibers [225]. The fibrin matrix also allows to produce strained fibrin gel by applying continuous tensile strain to fibrin scaffold. The morphological features of strained fibrin gels induce the alignment of seeded myoblasts. Moreover, the aligned cells are parallel to the direction of the strain reproducing the organization of skeletal muscle tissue [226]. The fibrin matrix also allows the differentiation of myoblasts, cultured in a three-dimensional pattern, under electrical stimulation [227]. Finally, fibrin scaffolds have been also combined with adult human cells to regenerate muscle after large tissue loss in a mouse model with large defect of tibialis anterior muscle. Constructs of fibrin microthreads and adult human cells were used, showing the role of constructs in host tissue regeneration by forming skeletal muscle-fibers, connective tissue, and PAX7 positive cells [228].

Another type of natural scaffold is a hyaluronan-based hydrogel that has been used to perform the delivery of either SCs or MPCs in a mice model. The construct SC-hydrogel showed more enhancement of regeneration process with a higher number of new myofibers than MPC-hydrogel or hydrogel alone. In the muscle receiving the SCs, there was a functional SC niche associated with neural and vascular networks [229].

The acellular muscle ECM has been also investigated in muscle tissue-engineering field. The acellular muscle scaffold was derived from the extensor digitorum longus muscle, and was injected with myoblasts. The constructs allowed cell survival and proliferation and showed longitudinal contractile force on electrical stimulation [230].

Each type of scaffold shows specific proprieties and peculiar advantages. The final goal of scaffold fabrication consists of promoting the proliferation of muscle stem cells, their differentiation, and parallel alignment to obtain a new skeletal muscle-like tissue [40, 84, 231–235]. The application of scaffold for regeneration of muscle tissue could represents an interesting approach particularly in the major trauma with large loss of tissue. In the majority of muscle injuries, the role of scaffold remains unclear and maybe not as important as in bone or cartilage regeneration. In fact, the skeletal muscle is characterized by different layer of connective tissue, such as endomysium, perimysium, and epimysium, which seems to be able to drive the regeneration of new muscle-fibers without the need of scaffold [12, 24, 87, 236–245]. Further studies are required to identify the best scaffold for skeletal muscle tissue engineering. However, the combination of available techniques could represent the right way to fabricate the ideal scaffold.

## 6. Conclusion

Skeletal muscle injuries are the most common injury in sport, occurring with direct and indirect mechanisms. Their effective management is a challenging issue in orthopaedic sport medicine because of the residual effects, such as severe long-term pain and physical disability. Skeletal muscle injuries cause time loss of activity and increased risk of recurrent injury. For these reasons, they constitute a health problem for athletes and an economic problem for clubs and sponsors. 

In most of the instances, current therapy consists of conservative management including RICE protocol and administration of NSAIDs or intramuscular corticosteroids. However, current management of muscle injuries does not often provide an optimal restore of preinjury status because of the fibrosis which occurs during the repair process of injured muscle. Experimental studies highlight the biological bases of muscle healing after contusion, strain, or laceration injury. This provides the rationale basis for new biological therapies, such as PRP and growth factors, cell-based therapy and tissue engineering [62, 70, 84, 233, 237, 243, 245]. Biological strategies may well be more favourable to healing. Although PRP application is encouraged, reasons for concern persist in its use for muscle injury management, and its mechanism of action remains uncertain. Further research is required to allow a standardized and safety use of this product in clinical practice [73]. Cell-based strategies have been investigated only in limited and inconsistent studies. The role of stem cells needs to be confirmed. Further research is required to identify mechanisms involved in muscle regeneration and in survival, proliferation, and differentiation of stem cells. Skeletal muscle tissue engineering represents a biological alternative for replacement of large tissue loss after severe damage, based on combination of adult or embryonic stem cells, factors or stimuli, and biomaterials. However, further studies are required to identify the best biomaterial to fabricate the ideal scaffold, the best cell source for scaffold seeding, and the role of growth factors and other stimuli used to functionalize the scaffold.
